# Associations of fruit, whole grain, and total energy intakes with gut microbiome diversity and composition

**DOI:** 10.29219/fnr.v67.9725

**Published:** 2023-12-05

**Authors:** Yixiao Wang, Keming Zhang, Linna Dai, Fengya Sun, Dan Wang, Sijia Meng, Jing Zhao, Yanfang Liu, Wanting Liu, Chunyan Li, Yuan Wang, Wenli Lu, Yun Zhu

**Affiliations:** 1 Department of Epidemiology and Biostatistics, School of Public Health, Tianjin Medical University, Tianjin, China; 2 First Teaching Hospital of Tianjin University of Traditional Chinese Medicine, Tianjin, China; 3 Tianjin Nankai Hospital, Tianjin, China

**Keywords:** gut microbiota, fruits, whole grains, total energy

## Abstract

**Background:**

The relationship between fruit, whole grain, and total energy consumption and the gut microbiome in the Chinese population remains unclear.

**Objective:**

We investigated the relationship between intakes of fruits, whole grains, and energy, and the diversity and composition of gut microbiota.

**Design:**

This cross-sectional study included 167 subjects aged 40-75 years who underwent colonoscopy at Nankai Hospital in Tianjin, China. Each of the participants completed a personal history questionnaire, a 7-day dietary record, and donated a fecal sample. The V3-V4 hypervariable region of the bacterial 16S rRNAgene was amplified and sequenced using Illumina Novaseq. The relationship between diet and gut microbiota was evaluated in terms of both the overall composition and the abundance of specific taxon.

**Results:**

Fruits intake was positively related to the abundance of Bacilli, Porphyromonadaceae, Streptococcaceae, *Parabacteroides, Streptococcus*, and Bilophila in fecal samples. Higher whole grains intake was associated with higher microbial diversity, as measured by Shannon, Simpson, and Chao1 indices. Specifically, there was a significant increase inthe relative abundance of Lachnospiraceae and a decrease in Actinobacteria with increased whole grains intake. Moreover, higher intake of total energy was associated with a lower abundance of *Anaerostipes* and a higher abundance of Lactobacillales and *Acidaminococcus*.

**Conclusion:**

Whole grains intake was positively associated with gut microbial diversity. Fruits and total energy intake were related to the abundance of specifictaxon (e.g., Bacilli and Acidaminococcus). These findings highlight the potential importance of dietary interventions for modulating gut microbiota composition and promoting overall health.

## Popular scientific summary

- Eating whole grains is linked to a more diverse and beneficial gut microbiota composition.
- Fruit and overall energy intake are associated with specific microbial groups, such as Bacilli and *Acidaminococcus*.
- Our data emphasize the importance of dietary changes in controlling the makeup of gut bacteria and cultivating a healthier gut environment.

The human intestinal tract harbors an extremely complex and large microbiota, with microorganisms adding up to around 100 trillion in number. This microbiota interacts with the host via various physiological processes, such as shaping the intestinal epithelium, aiding food digestion and energy metabolism, regulating host immune function, and maintaining intestinal homeostasis (1–3). Gut dysbiosis can lead to a range of health problems, including inflammatory bowel disease (4), obesity (5), diabetes (6,7) and autoimmune diseases (8).

The composition and diversity of the intestinal microbiota are dynamic and can be influenced by various factors, including host genetics, immune response, antibiotic use, exercise, and diet (9–11). Among the various dietary components, fruits and whole grains are considered to be particularly beneficial due to their high fiber content and associated prebiotic effects (12–14). Current research suggests that consuming fruits and whole grains is associated with a more diverse and beneficial gut microbiota composition. Specifically, fruits are rich in fiber, polyphenols, and other nutrients that can promote the growth of beneficial bacteria, such as *Bifidobacteria* and *Lactobacillus*, while inhibiting the growth of harmful bacteria (15). Whole grains, which contain fiber, resistant starch, and other prebiotics, have also been found to increase the abundance of beneficial bacteria in the gut (e.g. *Prevotella* and *Roseburia*) by attenuating oxidative stress and inflammatory responses in serum (16,17). In contrast, research has suggested that overconsumption of calories can lead to a less diverse and less healthy gut microbiome through several mechanisms, including promoting inflammation, altering gut motility, and inducing metabolic dysfunction (18).

Despite the existing research on the association of fruits, whole grains, and total energy intakes with gut microbiome diversity, many questions remain unanswered. For instance, although short-term trials have shown that fruits, whole grains, and total energy can affect gut microbiome composition, it is not clear how habitual intake of these dietary factors impacts the microbiome. Additionally, because food culture and microbiota structure can vary greatly across geographical regions (19,20), and Chinese dietary customs are distinct from those of other nations, reflecting unique cultural practices and beliefs regarding food selection, preparation, and consumption, it is crucial to explore the relationship between diet and the microbiome, specifically in the Chinese population. However, research in this area remains limited.

Therefore, this study explored the associations between fruits, whole grains, and total energy intakes with gut microbiome diversity and composition in a Chinese population. By shedding light on these associations, we may gain a better understanding of the role that diet plays in shaping the gut microbiome, which could have implications for the development of personalized dietary interventions to improve human health.

## Materials and methods

### Study participants

We conducted a cross-sectional study as part of the Colorectal Cancer Screening Project in Tianjin, China. Individuals who underwent colonoscopy at Tianjin Nankai Hospital between March 2021 and March 2022 and aged between 40 and 75 years were eligible for inclusion. We further excluded: 1) individuals who had taken prebiotics, probiotics, synbiotics, antibiotics, or undergone radiation therapy within the past three months; 2) women who were pregnant or lactating; 3) individuals with immune deficiencies; 4) individuals with chronic or acute gastrointestinal disorders requiring medical attention, such as acute gastroenteritis, peptic ulcer, or dysentery; 5) those with infections in other areas of the body; 6) those diagnosed with familial adenomatous polyposis; 7) individuals with malignant tumors or who had undergone colorectal surgery, except for non-melanoma skin cancer; and 8) individuals with behavioral constraints, cognitive impairment, or who were extremely frail and unable to participate in the study. This study was approved by the Ethics Committee at Tianjin Nankai Hospital. All 167 subjects included in the analysis provided informed consent prior to participating in the study.

### Data collection

#### Lifestyle and diet assessment

Participants completed a personal history questionnaire regarding their health and lifestyle and a 7-day food record. Nutrient and total energy intakes were calculated by multiplying the food intake (in grams) by the corresponding nutrient content per gram based on the 2017 China Food Composition List (Volume 1, Second Edition).

#### Stool samples collection

Subjects were given fecal collection tools and instructed on how to collect fecal samples. Fresh fecal samples were collected from participants, immediately snap-frozen in liquid nitrogen, and transported to the lab within 48 hours. These samples were stored at -80°C for DNA extraction and gut microbiota sequencing.

#### DNA extraction and sequencing

Total DNA of microorganisms in fecal samples was extracted using the Stool Genomic DNA Extraction Kit (Solarbio, China), and the extracted DNA products were stored at -80°C for backup. The quantity and quality of genomic DNA were measured using the NanoDrop Spectrophotometer (Thermo Fisher Scientific, Waltham, MA, USA). The V3-V4 region of the bacterial 16S rRNA gene was amplified using specific primers 341F (5’-CCTACGGGNGGCWGCAG-3’) and 805R (5’-GACTACHVGGGTATCTAATCC-3’). The PCR products were purified using AMPure XP Beads (Beckman Coulter, Indianapolis, IN), and the quantization was performed using the Qubit dsDNA HS Assay Kit. The Illumina Novaseq 6000 SP PE250 (DiaCarta, Inc) was used for sequencing.

The data of each sample were split from the original data according to barcode sequences and primer sequences, and controlled and filtered for quality. Low-quality sequences were identified based on the following criteria: sequences less than 150bp, average mass below 20, lines containing ambiguous orders and sequences greater than 8bp, and lines containing single nucleotide repeats. Chimeric sequences were further removed.

### Bioinformatics analysis

In the study, the Vsearch-1.11.1 software was used to assign effective tags to operational taxonomic units (OTUs) based on a similarity threshold of >97%. The representative sequences from each OTU were annotated using default parameters and the Green Gene database to generate lists of OTUs. The community composition of each sample was calculated at each taxonomic level (i.e. kingdom, phylum, class, order, family, genus, and species). The OTUs containing less than 0.001% of the total rows in all models were removed. QIIME (version 1.7.1) was used to calculate alpha diversity indices, including Shannon, Simpson, and Chao1, while beta diversity indices were calculated using weighted Unifrac (21,22). Principal coordinate analysis (PCoA) was performed using WGCNA, stat, and ggplot2 in R software (version 2.15.3). Differences in Unifrac were compared using T-test and the Monte Carlo permutation test. The PERMANOVA (Permutational multivariate analysis of variance) method (23) was used to evaluate intergroup differentiation of microbial community structure.

### Statistical analysis

Data analysis was performed using SAS 9.4 software. Subjects were divided into three groups (low, medium and high intake groups) based on their tertile intakes of whole grains, fruits and total energy, respectively. The Shapiro-Wilk test was used to test for normality. Normally distributed continuous variables were described using mean ± standard deviation (x ± s), and one-way ANOVA was used to compare groups. Non-normally distributed continuous variables were described using median and interquartile range, and the Kruskal-Wallis test was used for comparison between groups. Categorical variables were described using composition ratios, and the chi-square test was used to compare between groups. We examined the relationship between dietary intakes and alpha diversity metrics (Shannon, Simpson, and Chao1) using multivariable linear regression analysis. Factors associated with gut microbiota in the literature were assessed as potential confounders. For instance, higher body mass index was associated with reduced levels of *Alistipes finegoldii* and *Alistipes senegalensis*, while increased yogurt intake correlated strongly with higher abundance of *Leuconostoc mesenteroides* and *Lactococcus lactis* (24). Smoking was found to decrease the abundance of *parabobacterides distasonis* and *Lactobacillus* spp., while simultaneously increasing the levels of *Eggerthella lenta* (25,26). These variables underwent stepwise selection, with a significance threshold of *P* < 0.05 for inclusion in the final model. The final list of covariates comprised age, sex, body mass index, fried and barbecue food intake, tea intake, yogurt intake, probiotic drink intake, alcohol consumption, smoking, and drug use. The Kruskal-Wallis H test was used to compare the differences of microbial community abundance in different food intake groups at class, family, genus, order and phylum levels. A *P*-value of <0.05 was considered statistically significant.

## Results

There was no significant difference between groups with respect to age, BMI, waistline, fried and barbecue food consumption, tea consumption, yogurt and probiotic drink intake, and alcohol drinking (*P* > 0.05). However, current smokers were more likely to have a low fruit intake than non-smokers (*P* = 0.012). Individuals having higher family income tended to consume more fruits and total energy (Table 1).

**Table 1:** Characteristics of the study population (*N* = 167)

Characteristics	Low fruits intake (*n* = 47)	Moderate fruits intake (*n* = 68)	High fruits intake (*n* = 52)	*P*	Low whole grains intake (*n* = 60)	Moderate whole grains intake (*n* = 51)	High whole grains intake (*n* = 56)	*P*	Low total energy intake (*n* = 39)	Moderate total energy intake (*n* = 51)	High total energy intake (*n* = 64)	*P*
Age (year)	59.57 ± 1.14	62.56 ± 0.83	61.39 ± 1.09	0.133	61.43 ± 0.92	60.75 ± 1.14	61.73 ± 1.01	0.601	61.54 ± 1.10	60.49 ± 1.03	61.90 ± 0.92	0.713
Sex (%)				0.398				0.513				0.002
Men	27 (60.0)	28 (46.7)	25 (53.2)		31 (56.4)	25 (55.6)	24 (46.2)		13 (33.3)	24 (48.0)	43 (68.3)	
Women	18 (40.0)	32 (53.3)	22 (46.8)		24 (43.6)	20 (44.4)	28 (53.8)		26 (66.7)	26 (52.0)	20 (31.7)	
Family income (yuan/month)	6000 (5800)	8000 (7500)	8500 (4300)	0.032	7000 (5000)	6500 (5000)	7200 (5000)	0.569	6000 (6000)	8000 (5000)	8000 (4000)	0.042
Body mass index (kg/m^2^)	24.11 ± 0.50	23.77 ± 0.39	24.51 ± 0.39	0.586	23.83 ± 0.42	24.09 ± 0.44	24.38 ± 0.42	0.547	24.29 ± 0.52	23.67 ± 0.38	24.32 ± 0.41	0.402
Waistline (cm)	86.86 ± 1.56	84.97 ± 1.30	86.59 ± 1.48	0.594	86.18 ± 1.32	85.80 ± 1.61	86.02 ± 1.43	0.917	84.38 ± 1.50	84.08 ± 1.31	88.67 ± 1.38	0.070
Fried and barbecue food (%)				0.360				0.246				0.647
Hardly	23 (52.3)	38 (64.4)	26 (56.5)		32 (58.2)	22 (50.0)	33 (66.0)		22 (56.4)	26 (52.0)	39 (65.0)	
<1/week to 1/week	17 (38.6)	17 (28.8)	17 (37.0)		20 (38.4)	17 (38.6)	14 (28.0)		14 (35.9)	22 (44.0)	15 (25.0)	
≥2–3 times/week	4 (9.1)	4 (6.8)	3 (6.5)		3 (5.4)	5 (11.4)	3 (6.0)		3 (7.7)	2 (4.0)	6 (10.0)	
Tea intake (%)				0.885				0.471				0.431
Hardly	16 (36.4)	26 (44.1)	15 (32.6)		24 (43.6)	13 (29.5)	20 (40.0)		16 (41.0)	19 (38.0)	22 (36.7)	
<1/week to 2–3 times/week	9 (20.4)	9 (15.2)	13 (28.3)		10 (18.2)	12 (27.3)	9 (18.0)		5 (12.8)	14 (28.0)	12 (20.0)	
≥4–6 times/week	19 (43.2)	24 (40.7)	18 (39.1)		21 (38.2)	19 (43.2)	21 (42.0)		18 (46.2)	17 (34.0)	26 (43.3)	
Yogurt and probiotic drink intake (%)				0.567				0.761				0.470
Hardly	28 (61.9)	27 (45.8)	22 (47.8)		31 (55.5)	20 (44.2)	26 (52.0)		24 (61.5)	20 (40.0)	33 (55.0)	
<1/week to 2–3 times/week	12 (28.6)	25 (42.4)	17 (37.0)		17 (31.5)	19 (44.2)	18 (36.0)		12 (30.8)	23 (46.0)	19 (31.7)	
≥4-6 times/week	4 (9.5)	7 (11.8)	7 (15.2)		7 (13.0)	5 (11.6)	6 (12.0)		3 (7.7)	7 (14.0)	8 (13.3)	
Insoluble fiber intake (g/day)	8.00 (6.85)	10.86 (4.36)	13.45 (10.17)	0.000015	9.43 (6.77)	10.62 (5.65)	12.65 (7.63)	0.002	7.45 (4.37)	10.72 (6.32)	12.89 (6.27)	0.001
Drink (%)				0.281				0.525				0.770
Non-drinkers	23 (52.27)	38 (64.41)	30 (65.22)		31 (56.36)	25 (56.82)	35 (70.00)		26 (66.67)	35 (70.00)	30 (50.00)	
Current drinkers	16 (36.36)	15 (25.42)	8 (17.39)		16 (29.09)	14 (31.82)	9 (18.00)		11 (28.21)	11 (22.00)	17 (28.33)	
Ex-drinkers	5 (11.36)	6 (10.17)	8 (17.39)		8 (14.55)	5 (11.36)	6 (12.00)		2 (5.13)	4 (8.00)	13 (21.67)	
Smoking (%)				0.012				0.546				0.618
Non-smokers	27 (61.36)	48 (81.36)	31 (67.39)		39 (70.91)	31 (70.45)	36 (72.00)		31 (79.49)	37 (74.00)	38 (63.33)	
Current Smokers	13 (29.55)	7 (11.86)	5 (10.87)		12 (21.82)	6 (13.64)	7 (14.00)		6 (15.38)	7 (14.00)	12 (20.00)	
Ex-smokers	4 (9.09)	4 (6.78)	10 (21.74)		4 (7.27)	7 (15.91)	7 (14.00)		2 (5.13)	6 (12.00)	10 (16.67)	
Drug use (%)				0.105				0.939				0.043
Yes	24 (54.5)	44 (74.6)	30 (65.2)		37 (67.3)	29 (65.9)	32 (64.0)		25 (64.1)	27 (54.0)	46 (76.7)	
No	20 (45.5)	15 (25.4)	16 (34.8)		18 (32.7)	15 (34.1)	18 (36.0)		14 (35.9)	23 (46.0)	14 (23.3)	

Whole grain intake (g/day): 1^st^ tertile (≤64.5), 2^nd^ tertile (64.5-125.5), 3^rd^ tertile (>125.5);

Fruit intake (g/day): 1^st^ tertile (≤100.0), 2^nd^ tertile (100.0-217.9), 3^rd^ tertile (>217.9);

Total energy intake (kcal/day): 1^st^ tertile (≤1637.5), 2^nd^ tertile (1637.5-2014.9), 3^rd^ tertile (>2014.9)

Normally distributed continuous variables were described using mean ± standard deviation (x ± s)

Non-normally distributed continuous variables were described using median and interquartile range

Categorical variables were presented as percentage.

Drug use included medications for gastropathy, hypertension, hyperlipidemia, diabetes, hyperuricemia, and non-steroidal anti-inflammatory drugs etc.

### α-Diversity indices by food intakes

The Shannon, Simpson, and Chao1 indices were used to assess the species richness and diversity in different intake groups (Table 2). The results demonstrated that there were significant differences in the diversity of the gut microbiota across the whole grains intake groups, with the high whole grains intake group having the highest species richness (*P* for Shannon diversity index = 0.0381; *P* for Chao1 diversity index = 0.0260). No significant differences were found in any of the diverse indices across groups defined by fruits and total energy intakes.

**Table 2:** Comparison of α diversity of intestinal microbiota across three groups

Dietary intakes	No. of participants	Shannon		Simpson		Chao1	
		β (95% CI)	*P*	β (95% CI)	*P*	β (95% CI)	*P*
Fruits intake							
Low	47	ref		ref		ref	
Moderate	68	0.063 (-0.199~0.326)	0.634	0.00007 (-0.00020~0.00033)	0.622	-123.614 (-1153.709~906.481)	0.813
High	52	-0.043 (-0.312~0.226)	0.750	0.00007 (-0.00034~0.00020)	0.621	-165.042 (-1219.370~889.286)	0.757
Whole grains intake							
Low	60	ref		ref		ref	
Moderate	51	0.196 (-0.052~0.445)	0.120	0.00017 (-0.00009~0.00042)	0.198	674.686 (-301.509~1650.880)	0.174
High	56	0.305 (0.063~0.546)	0.014	0.00026 (0.00001~0.00050)	0.040	1030.050 (80.262~1979.838)	0.034
Total energy intake							
Low	39	ref		ref		ref	
Moderate	51	0.169 (-0.104~0.442)	0.222	0.00020 (-0.00007~0.00048)	0.150	396.859 (-667.996~1461.714)	0.462
High	64	0.058 (-0.213~0.328)	0.675	0.00004 (-0.00023~0.00032)	0.750	-316.585 (-1373.670~740.500)	0.555

Whole grain intake (g/day): 1^st^ tertile (≤64.5), 2^nd^ tertile (64.5-125.5), 3^rd^ tertile (>125.5);

Fruit intake (g/day): 1^st^ tertile (≤100.0), 2^nd^ tertile (100.0-217.9), 3^rd^ tertile (>217.9);

Total energy intake (kcal/day): 1^st^ tertile (≤1637.5), 2^nd^ tertile (1637.5-2014.9), 3^rd^ tertile (>2014.9)

Continuous variables were described using mean ± standard deviation (x ± s)

Multivariable linear models adjusted for age, sex, body mass index, fried and barbecue food intake, tea intake, yogurt intake, probiotic drink intake, alcohol consumption, smoking and drug use (e.g. medications for gastropathy, hypertension, hyperlipidemia, diabetes, hyperuricemia, and non-steroidal anti-inflammatory drugs).

### β-Diversity by food intakes

**Fig. 1:**
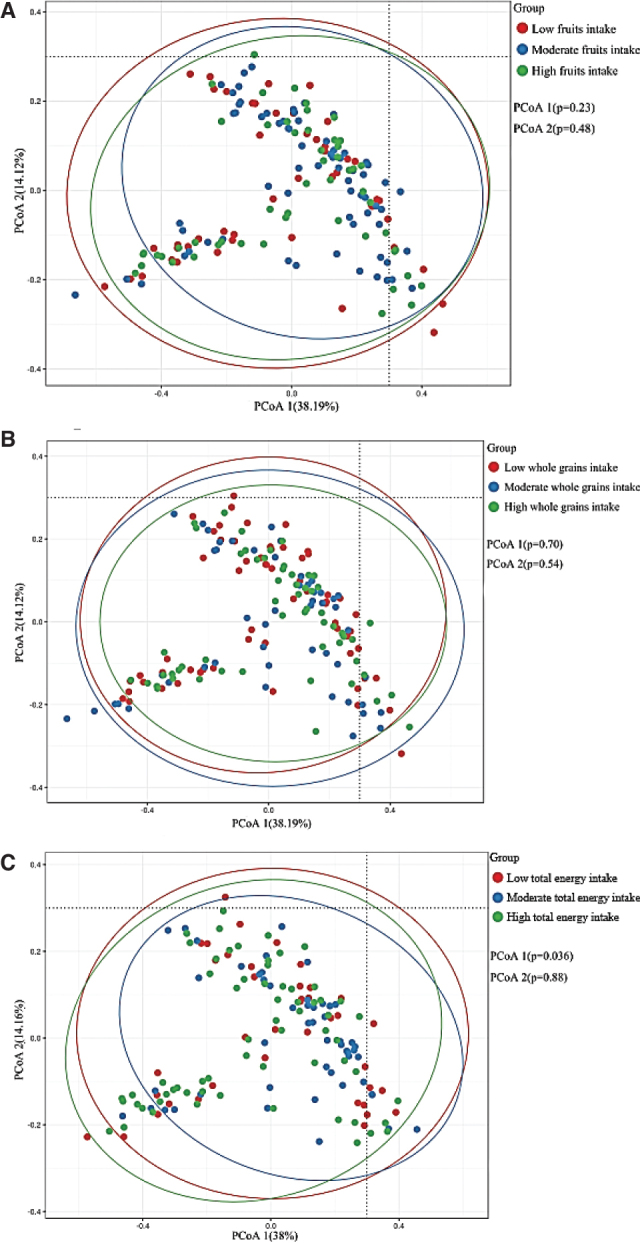
Variation in the gut microbiota composition represented by unconstrained PCoA based on the distance indexes (A–C).

The Weighted Unifrac Distances analysis detected compositional dissimilarities (β-diversity) of the gut microbiota across different groups defined by total energy intake (Figure 1C, *P* for PCoA1 = 0.036), although no apparent clustering was observed among the groups defined by whole grains and fruits intakes.

### Associations between dietary intakes and relative abundances of taxa

Our findings indicated that individuals with increased fruits intake demonstrated a marked increase in Bacilli richness at the class level and in Porphyromonadaceae and *Streptococcus* richness at the family level. At the genus level, the richness of Parabacteroides, *Streptococcus*, and *Bilophila* increased significantly, consequent to augmented fruits consumption (Table 3).

**Table 3:** Differences of microbial community abundance across three groups at class, family, genus, order and phylum levels

Level	microflora	Low fruits intake (*n* = 47) (%)	Moderate fruits intake (*n* = 68) (%)	High fruits intake (*n* = 52) (%)	*P*	Low whole grains intake (*n* = 60) (%)	Moderate whole grains intake (*n* = 51) (%)	High whole grains intake (*n* = 56) (%)	*P*	Low total energy intake (*n* = 39) (%)	Moderate total energy intake (*n* = 51) (%)	High total energy intake (*n* = 64) (%)	*P*
Phylum	Firmicutes	46.74	55.53	46.47	0.0052	45.39	49.60	52.18	0.0753	46.35	51.20	48.37	0.2931
	Bacteroidetes	41.23	37.29	44.75	0.0491	43.24	41.90	40.76	0.6091	44.63	40.20	41.99	0.2587
	Actinobacteria	2.75	1.40	2.05	0.4393	2.28	1.95	1.76	0.0295	1.58	1.83	2.62	0.7644
Class	Gammaproteobacteria	5.41	4.29	4.44	0.0345	6.58	4.20	2.99	0.2719	6.62	3.21	4.53	0.6880
	Bacilli	0.36	0.51	0.63	0.0344	0.62	0.65	0.24	0.8448	0.33	0.15	0.89	0.5888
	Actinobacteria	1.28	1.65	2.27	0.8281	2.07	1.59	1.51	0.0221	2.61	1.12	1.67	0.4603
	Clostridia	45.85	50.51	47.60	0.2843	44.58	48.83	51.79	0.0593	46.24	55.23	45.43	0.0034
	Bacteroidia	44.63	40.20	41.99	0.2587	43.24	41.90	40.76	0.6091	41.23	37.29	44.75	0.0321
Order	Clostridiales	46.24	55.23	45.43	0.0034	44.58	48.82	51.79	0.0577	45.85	50.51	47.60	0.2843
	Bifidobacteriales	1.25	1.62	2.26	0.8377	2.04	1.57	1.49	0.0332	2.60	1.11	1.64	0.5071
	Bacteroidales	44.63	40.20	41.99	0.2587	43.24	41.90	40.76	0.6091	41.23	37.29	44.75	0.0321
	Enterobacteriales	6.42	3.09	4.08	0.6843	6.12	3.99	2.72	0.2514	4.84	4.12	4.14	0.0301
	Lactobacillales	0.31	0.13	0.88	0.4419	0.61	0.63	0.22	0.5439	0.35	0.48	0.61	0.0347
Family	Ruminococcaceae	15.01	20.93	18.58	0.0395	15.84	19.57	20.47	0.0959	19.07	21.87	16.85	0.0562
	Enterobacteriaceae	4.84	4.12	4.14	0.0301	6.12	3.99	2.72	0.2514	6.42	3.09	4.08	0.6843
	Porphyromonadaceae	0.81	1.37	1.38	0.0170	1.04	1.43	1.21	0.5895	1.03	1.43	1.02	0.4151
	Barnesiellaceae	0.28	0.66	0.17	0.0391	0.43	0.44	0.34	0.6722	0.72	0.41	0.26	0.0936
	Streptococcaceae	0.30	0.37	0.54	0.0141	0.49	0.56	0.15	0.3860	0.25	0.12	0.71	0.3913
	Lachnospiraceae	19.15	17.48	17.27	0.9309	16.05	16.70	20.92	0.0167	16.15	21.93	16.77	0.0100
	Bifidobacteriaceae	1.25	1.62	2.26	0.8377	2.04	1.57	1.49	0.0332	2.60	1.11	1.64	0.5071
	*Parabacteroides*	0.81	1.37	1.38	0.0170	1.04	1.43	1.21	0.5873	1.04	1.44	1.02	0.4110
	*Klebsiella*	0.77	0.35	0.65	0.0034	0.59	0.48	0.61	0.8139	0.52	0.42	0.76	0.7860
	*Streptococcus*	0.30	0.37	0.54	0.0141	0.49	0.56	0.15	0.3860	0.25	0.12	0.71	0.3913
	*Bilophila*	0.11	0.16	0.20	0.0402	0.14	0.16	0.17	0.8699	0.19	0.15	0.14	0.5746
Genus	*Roseburia*	2.77	1.52	1.88	0.1919	1.33	2.31	2.37	0.0122	1.56	2.75	1.75	0.2093
	*Bifidobacterium*	1.25	1.62	2.26	0.8377	2.04	1.57	1.49	0.0332	2.60	1.11	1.64	0.5071
	*Anaerostipes*	0.10	0.16	0.10	0.0773	0.08	0.21	0.09	0.1072	0.15	0.11	0.08	0.0220
	*Acidaminococcus*	/	/	/	/	/	/	/	/	0.04	0.13	0.18	0.0346

Whole grain intake (g/day): 1^st^ tertile (≤64.5), 2^nd^ tertile (64.5-125.5), 3^rd^ tertile (>125.5);

Fruit intake (g/day): 1^st^ tertile (≤100.0), 2^nd^ tertile (100.0-217.9), 3^rd^ tertile (>217.9);

Total energy intake (kcal/day): 1^st^ tertile (≤1637.5), 2^nd^ tertile (1637.5-2014.9), 3^rd^ tertile (>2014.9)

*P* values were calculated from the Kruskal-Wallis H test.

While the relative abundances of Bacteroidetes, Firmicutes, Gammaproteobacteria, Clostridiales, Barnesiellaceae, Ruminococcaceae, Enterobacteriaceae, and *Klebsiella* exhibited significant intergroup differences, no discernible dose-response relationship with fruits intake was observed.

Similarly, increased whole grains intake was associated with a significant decrease in Actinobacteria abundance at both phylum and class levels and in *Bifidobacterium* abundance at the order, family and genus levels. Conversely, we observed an overrepresentation of Lachnospiraceae at the family level and an increase of *Roseburia* at the genus level with increased whole grains consumption.

As for energy intake, an increase in the abundance of Lactobacillales and *Acidaminococcus*, along with a decrease in the richness of *Anaerostipes*, was observed with increased total energy intake. There were no significant differences in the relative abundance of other taxa.

## Discussions

Our data indicated that the consumption of whole grains was positively associated with greater gut microbial diversity, whereas fruits intake and total energy intake appeared to have a more limited effect on microbial diversity. Additionally, an increase in the intake of whole grains was associated with a significant rise in the relative abundance of Lachnospiraceae and *Roseburia*, as well as a decrease in Actinobacteria and *Bifidobacterium*. A higher intake of fruits had a positive correlation with the abundance of Bacilli, Porphyromonadaceae, Streptococcaceae, *Parabacteroides*, *Streptococcus*, and *Bilophila* in fecal samples. On the other hand, a higher intake of total energy appeared to be linked with a lower abundance of *Anaerostipes*, and a higher abundance of Lactobacillales and *Acidaminococcus*.

Recent studies have investigated the relationship between whole grains intake and gut microbiota diversity and abundance, with varying results. Consistent with the findings of our data, most studies (27–30), but not all (31), found that individuals who consume whole grains have a more diverse gut microbiota. Consuming whole grains promotes a more diverse gut microbiota due to the variety of complex carbohydrates and nutrients (e.g. dietary fiber and prebiotics) present in whole grains, which provide substrates for the growth of diverse bacterial species in the gut.

In terms of gut microbiota abundance, our findings are in line with an earlier study of 1717 individuals with diverse ethnic backgrounds, in which the consumption of whole grains products was associated with a reduction in the relative abundance of Actinobacteria at the phylum level, in contrast to refined grains intake (32). The effect of Actinobacteria on health varies depending on the context. While some species, such as *Mycobacterium tuberculosis*, *Corynebacterium diphtheriae*, and *Tropheryma whipplei*, are associated with diseases like tuberculosis, diphtheria, and Whipple’s disease (33), most Actinobacteria have positive effects; *Bifidobacteria*, particularly, are widely used as probiotics and show beneficial effects in various pathological conditions. Additionally, our study replicated prior findings demonstrating a noticeable increase in the abundance of *Roseburia* at the genus level subsequent to increased whole grains consumption (32). Evidence suggests that *Roseburia* has a positive influence on inflammatory bowel disease, alcoholic fatty liver, colorectal cancer, and metabolic syndrome, positioning it as a potential ‘Next Generation Probiotic’ (34).

Existing small-scale whole grains intervention trials yield inconclusive findings. The majority of intervention trials indicated a correlation between the consumption of whole grains and elevated levels of certain beneficial gut bacteria, such as *Bifidobacterium* (35–37), *Lactobacillus* (35) and Firmicutes (38). Likewise, Vitaglione et al. demonstrated an increase in Prevotella levels following an intervention involving whole grain consumption (39). In contrast, another randomized crossover study conducted on 33 healthy individuals with low habitual whole grain consumption did not replicate these findings. That study reported that increased intake of whole grains had no significant influence on the abundance of gut microbiota (40). Despite prior evidence suggesting a positive relationship between the intake of whole grains and the abundance of *Bifidobacterium* and *Prevotella*, the present study did not yield any such association. It is worth noting that differences in study design, sample size, methods for analyzing gut microbiota, and the compositional complexity of whole grains, which contain a variety of carbohydrates, can all contribute to varying results in these types of studies. Further investigation is necessary to comprehensively elucidate the correlation between consumption of whole grains and the diversity and abundance of gut microbiota, particularly through conducting large-scale clinical trials.

Multiple studies have shown that increased fruits intake is associated with increased gut microbial diversity and abundance. Our findings regarding the link between fruits consumption and gut microbiota are consistent with prior research which has indicated that the polyphenols found in plant-based diets can promote the growth of beneficial bacteria, including *Porphyromonas*, while also regulating the balance of gut microbes and hindering the growth of harmful bacteria (41,42). A study on 37 well-nourished Australian children aged between 2 and 3 years exhibited that *Prevotella* increases with the consumption of a plant-based diet (43). However, in our study, we could not find any significant relationship between this flora and fruits consumption. Conversely, that study displayed a negative correlation between fruits intake and the relative abundance of bacteria linked to *Ruminococcus gnavus*.

The mechanisms underlying the association between fruits intake and gut microbiota are not yet fully understood. Fruits consumption may impact gut bacteria by providing prebiotic fibers, which can stimulate the growth and function of beneficial bacteria (44,45). In addition, gut bacteria can produce short-chain fatty acids (SCFAs) through the fermentation of dietary fibers, which have multiple health benefits, including reducing inflammation and improving gut barrier function (46,47). Some evidence suggests that fruits intake may enhance SCFA production by fostering the growth of fiber-fermenting gut bacteria. Further research is required to determine the specific types and amounts of fruits that are most advantageous for gut health and to comprehensively comprehend the underlying mechanisms.

Elevated calorie consumption, particularly from diets high in fat and sugar, has the potential to alter the composition of gut microbiota, leading to a decline in beneficial bacteria and an increase in harmful ones. A study in mice found that a high-calorie ‘Western’ diet decreased Bacteroidetes and increased Proteobacteria and Firmicutes. The effects became stronger with higher diet intake (48). In the current investigation, an increase in total energy intake was associated with a rise in Lactobacillales abundance at the order level. At the genus level, the richness of *Anaerostipes* declined, whereas the richness of *Acidaminococcus* increased following high total energy consumption. These findings are noteworthy, as Lactobacillales and *Acidaminococcus* have been identified as significant contributors to sleep disorders (49) and immune-related adverse events, respectively (50). Contrarily, an increased presence of *Anaerostipes* has been associated with a protective effect against certain health conditions, including Cushing’s syndrome (51). In a randomized controlled trial involving overweight/obese men and women with metabolic syndrome, the experimental group with restricted energy intake following a Mediterranean diet pattern experienced changes in their intestinal microbiota, including a decrease in *Butyricicoccus*, while *Haemophilus, Ruminiclostridium*, and *Eubacterium hallii* increased. Nonetheless, our study did not reveal any significant associations or trends between total energy intake and the aforementioned flora; further research is required to elucidate the specific mechanisms involved (52).

Furthermore, alterations in gut microbiota beta diversity have been observed in several diseases, including inflammatory bowel disease, irritable bowel syndrome, obesity, metabolic syndrome, and mental health disorders (53–55). However, it remains uncertain whether and how the distinct microbial clusters influenced by total energy intake are linked to individual health, necessitating further investigation. It should be noted that alterations in beta diversity alone may not directly trigger these conditions, but instead, they may indicate dysbiosis or imbalances in the gut microbiota.

Our study was characterized by strengths that involved the utilization of 7-day dietary diaries, which offered several advantages over food questionnaires in capturing food consumption, including more detailed and accurate recording of intake over several days, allowing for a more detailed analysis of nutrient content, and providing a more accurate measure of energy intake. The cross-sectional design of our study precluded establishing causality or assessing diet-induced changes in the microbiome. To address these limitations, a longitudinal study with a long-term follow-up is required. Future research directions should involve investigating the impact of specific food items or nutrients on the composition of the gut microbiota. It is recommended that these studies adopt a longitudinal approach, incorporate larger sample sizes, and employ more precise nutrient assessment techniques, such as the utilization of blood biomarkers.

## Conclusions

The consumption of whole grains demonstrated a positive correlation with gut microbial diversity, whereas the intake of fruits and overall energy was found to be associated with the prevalence of specific taxonomic groups such as Bacilli and *Acidaminococcus*. These observations underscore the potential significance of dietary interventions in regulating the composition of gut microbiota and fostering a healthy gut microbiome. This knowledge can potentially lead to the development of dietary recommendations tailored to the Chinese population.

## Informed consent statement

Written informed consent was obtained from all study participants.

## Conflict of interest and funding

The authors declare no conflict of interest. This research was supported by the National Natural Science Foundation of China (grant No. 82003533) and the CNS-ZD Tizhi and Health Fund (grant No. CNS-ZD2020-82).

## Authors’ contributions

Conceptualization, Y.Z., WL.L., and Y.W.; methodology, Y.Z., WL.L., and Y.W.; software, YX.W.; validation, Y.W., J.Z., L.D., Y.Z.; formal analysis, Y.W.; investigation, Y.Z., WL.L., and Y.W.; resources, WL.L., C.L. and K.Z.; data curation, YX.W., L.D., F.S., D.W., S.M., J.Z., Y.L., W.L., K.Z.; writing—original draft preparation, YX.W., L.D., F.S., J.Z., Y.L.; writing—review and editing, YX.W., L.D., F.S., D.W., S.M., J.Z., Y.L.; Y.Z., W.L., and Y.W.; supervision, Y.Z., C.L., K.Z., WL.L., and Y.W.; project administration, Y.Z., WL.L., and Y.W.; funding acquisition, Y.Z. All authors have read and agreed to the published version of the manuscript.
